# Case Report of a Large Ovarian Cyst in a Multiparous Woman: A Challenging Surgical Management

**DOI:** 10.1155/crog/2109088

**Published:** 2026-03-08

**Authors:** Ibrahim Talat Kasar, Atheer Hameed Alqarni, Elhadi Miskeen, Anwar Ali Alshehri, Naif Abdulaziz Alqarni, Mirnan Adel Alghamdi, Amal Fayez Hamuman, Amal Ahmed Alotaibi, Tagwa Idris, Laila Yahya Alhubaishi

**Affiliations:** ^1^ Department of Obstetrics and Gynecology, Maternal and Child Hospital, Bisha, Saudi Arabia; ^2^ Department of Obstetrics and Gynecology, College of Medicine, University of Bisha, Bisha, Saudi Arabia, ub.edu.sa; ^3^ College of Medicine, Imam Mohammad Ibn Saud Islamic University, Riyadh, Saudi Arabia, imamu.edu.sa; ^4^ Massachusetts General Hospital, Boston, Massachusetts, USA, harvard.edu; ^5^ Department of Obstetrics and Gynecology, College of Medicine and Health Sciences, Mohammed Bin Rashid University, Dubai, UAE, mbruniversity.ac.ae

**Keywords:** benign ovarian cyst, cystectomy, large ovarian cyst, multilocular cyst, ovarian cysts, ovarian neoplasms, salpingo-oophorectomy, symptomatic ovarian cyst

## Abstract

This case report details the surgical management of a large (15 × 9 cm) symptomatic right ovarian cyst in a 42‐year‐old multiparous woman, highlighting the decision‐making process for definitive treatment. The patient presented with chronic pelvic pain and a palpable mass, significantly reducing her quality of life. Imaging revealed a large, multilocular, benign‐appearing cystic lesion. Given the patient′s age, completion of childbearing, and the cyst′s size and complexity, a right salpingo‐oophorectomy was performed instead of a cystectomy to achieve definitive symptom resolution and mitigate recurrence risk. The decision to proceed with a definitive right salpingo‐oophorectomy, rather than an ovarian cystectomy, was based on a confluence of factors: the patient′s age (42), her status as a multiparous woman who had completed childbearing, and the cyst′s size and complex multilocular morphology which increased the technical difficulty and potential morbidity of cystectomy while raising a low‐grade concern for underlying neoplasia. The surgery was uncomplicated, and histopathology confirmed a benign cystadenoma. This case underscores the importance of tailoring surgical intervention, specifically the choice between cystectomy and salpingo‐oophorectomy, to individual patient factors such as parity, fertility goals, and cyst characteristics to optimize clinical outcomes.

## 1. Introduction

Ovarian neoplasm causes an adequate and complex problem in the health of women. Most ovarian masses are functional cysts and other benign tumors [1–3]. The global incidence of ovarian cysts is substantial, with estimates exceeding 16.7%, exhibiting a notable increase in prevalence during the reproductive years [4–6]. Ovarian neoplasms present with a very diverse range of symptoms, from fully asymptomatic instances to those characterized by incapacitating symptoms that significantly impair quality of life [7, 8]. Pelvic pain, a frequently reported symptom, can be either acute or chronic, varying in intensity from slight discomfort to excruciating, debilitating pain [9, 10]. Common complaints include bloating and abdominal distension, which are commonly linked to a sense of fullness or pressure in the lower abdomen. These symptoms, which may interfere with everyday activities and have an impact on a woman′s overall health, can be persistent or intermittent [11, 12]. Ultrasound is often the first imaging test since it is easy to get, is cheap, and can tell the difference between cystic and solid parts. MRI, on the other hand, often gives a more thorough picture of the mass′s features, such as how blood flows through it and how it relates to nearby organs [13, 14]. An extensive detail of the case tries to increase the understanding of ovarian cysts, their diverse manifestations, and therapeutic approaches, eventually promoting better patient results. The case reports a 42‐year‐old female patient with a large ovarian cystic tumor, requiring a complex cystectomy.

## 2. Case Report

A 42‐year‐old multiparous woman (para 5), with a prior cesarean delivery, presented with chronic right pelvic pain and pelvic heaviness for a long time. The pain was accompanied by discomfort and did not radiate anywhere; it also was not relieved by medication. The patient denied any abnormal bleeding. The patient had no vomiting, diarrhea, or other gastrointestinal symptoms. She had her menarche at the age of 14, and her menstrual cycles were regular. She used to take hormonal contraceptives previously. She had no medical or other surgical conditions, except her C‐section (CS), was not on any medication now, and did not have any relevant familial medical history. With a body mass index (BMI) of 28, all her vital signs were within the normal range. An abdominal examination showed a visible right iliac fossa mass. Relatively large on palpation, it was slightly movable and not fixed, and there was no guarding of the abdomen. A complete blood count showed that the patient had a white blood cell count of 7.84 × 103/*μ*L, a hemoglobin level of 13.5 g/dL, and a platelet count of 270 × 103/*μ*L. The patient underwent an ultrasound, which showed a right adnexal simple cyst measuring 10 × 5 cm. There were no visible intrauterine gestational sacs or any masses.

**Figure 1:**
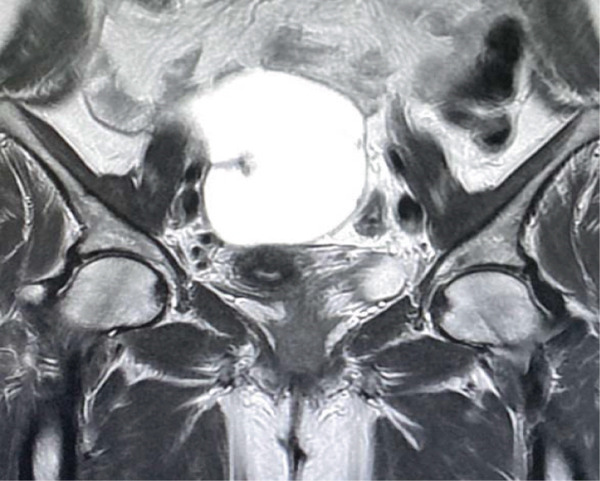
MRI with contrast showed a large, well‐defined right adnexal/ovarian cystic lesion measuring 12 × 10 × 6 cm.

**Figure 2:**
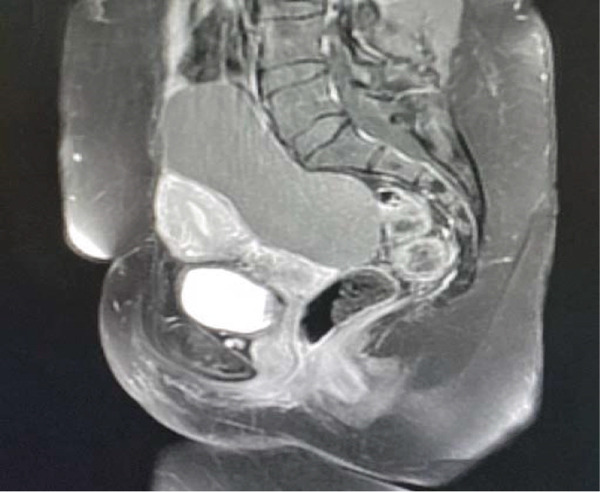
MRI postcontrast showed multilocular appearances with a few smooth septations, which showed postcontrast thin enhancement.

For detailed characterization, an MRI was performed, confirming a well‐defined right adnexal cystic lesion measuring 12 × 10 × 6 cm. The lesion was predominantly fluid‐filled, exhibiting low T1 and high T2 signal intensity. Its internal architecture was multilocular with a few thin, smooth septations, and it contained a small focus of high T1 and low T2 signal—not suppressed on fat‐saturated sequences—consistent with old hemorrhage. Postcontrast images demonstrated only thin, mild septal enhancement, with no evidence of a solid enhancing nodule, irregular/thickened septations (> 3 mm), or necrosis. Anatomically, the cyst was well‐demarcated from adjacent pelvic structures without signs of invasion. Based on these features—simple fluid content, smooth thin septations, and absence of solid components—the lesion was radiologically assessed as a benign complex cyst, such as a cystadenoma or hemorrhagic cyst (Figures 1 and 2).

**Figure 3:**
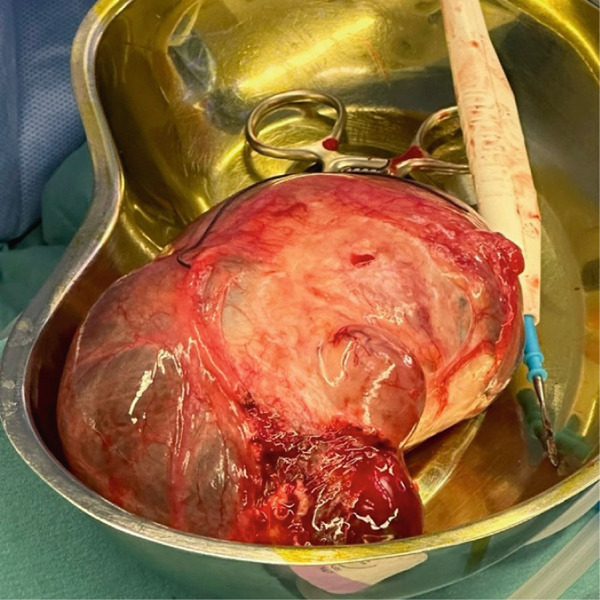
Intraoperative picture showing a large ovarian cyst.

**Figure 4:**
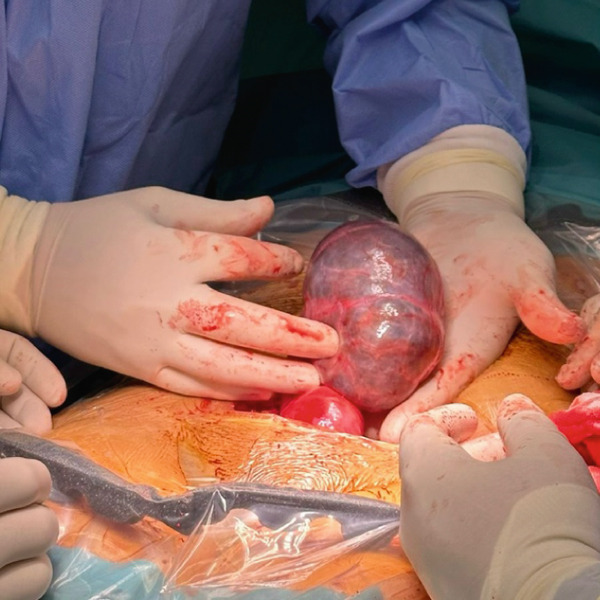
Intraoperative picture showing a large ovarian cyst during ovarian cystectomy.

Given the patient′s premenopausal status and the classic benign imaging features on MRI, the preoperative workup did not include serum tumor markers (e.g., CA‐125), as they can be nonspecifically elevated in benign conditions and were not deemed necessary for clinical decision‐making. The patient′s consent was obtained regarding the operation for right ovarian cystectomy or hysterectomy if the operation was complicated by adhesions or bleeding. Under general anesthesia, a right salpingo‐oophorectomy was performed on the patient (Figure 3). Following the removal of the ovarian cyst, the infundibulopelvic ligament was tied off. Figure 4 shows that the ovarian pedicle was cut and sutured without any issues or adhesions. After the surgery, the patient recovered well and experienced no problems. The histopathological analysis indicated a convoluted cyst wall of luteinized granulosa cells, accompanied by an inner fibrous tissue layer and an outer layer of theca cells. The attached fallopian tube appeared normal, and the tissues showed signs of corpora albicans. There were no signs of malignancy in the examined section, which offered reassurance and hope regarding the patient′s future health. No evidence of malignancy could be detected in the section examined, providing relief and optimism about the patient′s future health. An intact cyst measured 15 × 9 cm, with a smooth, thin wall, filled with clear yellow fluid, with a stretched tube measuring 10 cm in length, and Rep. sections are submitted in three cassettes. The patient was scheduled for follow‐up appointments 1 month and 3 months after her surgery. During both visits, she reported a full recovery from her previous symptoms.

## 3. Discussion

The case reports a 42‐year‐old woman with a large, symptomatic right ovarian cyst (12 × 10 × 6 cm on MRI and 15 × 9 cm on surgical measurement). Given the patient′s age (42), multiparous status with completed childbearing, and the cyst′s large size and complex multilocular morphology, a definitive right salpingo‐oophorectomy was performed instead of ovarian cystectomy. This approach prioritized complete resolution of symptoms, eliminated recurrence risk, and provided definitive histopathological diagnosis, balancing surgical feasibility with the patient′s clinical context. A critical aspect of this case was the surgical decision to perform a right salpingo‐oophorectomy instead of an ovarian cystectomy. The primary clinical challenge was the cyst′s large size, which presented a fairly surgical challenge. The patient′s chronic pelvic pain and heaviness, despite the absence of other alarming symptoms such as abnormal bleeding or gastrointestinal distress, highlight the importance of thorough investigation of persistent pelvic complaints even in the absence of overtly concerning signs. Several studies highlight the surgical challenge in managing large ovarian cysts [15, 16]. While giant cysts (often defined as > 20 cm) present heightened risks of complications and technical difficulty [17, 18], large cysts like the one in this case still represent a significant surgical consideration due to their mass effect and complex morphology. The successful removal without complications was due to careful surgical techniques. In comparison to other reported cases of large ovarian cysts, this case demonstrates a successful outcome with minimal morbidity. Reports in literature often describe examples of important complications, especially with large or more complex cysts [19, 20]. The absence of intraoperative or postoperative complications emphasizes careful preoperative planning, careful surgical technology, and the importance of appropriate patient selection for the surgical process.

The preoperative MRI was pivotal in risk stratification and surgical planning. Applying the ovarian–adnexal reporting and data system (O‐RADS) MRI risk score, the lesion′s features—a predominantly cystic structure with smooth, thin septations and no solid tissue—are consistent with an O‐RADS 2 classification (almost certainly benign). This standardized assessment provides a high level of confidence in excluding malignancy preoperatively, which was instrumental in opting for a minimally invasive surgical approach and discussing conservative versus definitive surgical options with the patient [21].

In this case, the management approach aligns with contemporary guidelines for adnexal masses, which emphasize risk stratification to guide intervention. Key recommendations from the American College of Obstetricians and Gynecologists and the Society of Gynecologic Oncologists highlight the use of imaging patterns and, where indicated, tumor markers to triage lesions and define surgical urgency [22]. In this case, the benign O‐RADS MRI features permitted classification as a low‐risk symptomatic mass, appropriately directing management toward elective definitive surgery rather than urgent oncologic resection, consistent with guideline‐based care.

**Figure 5:**
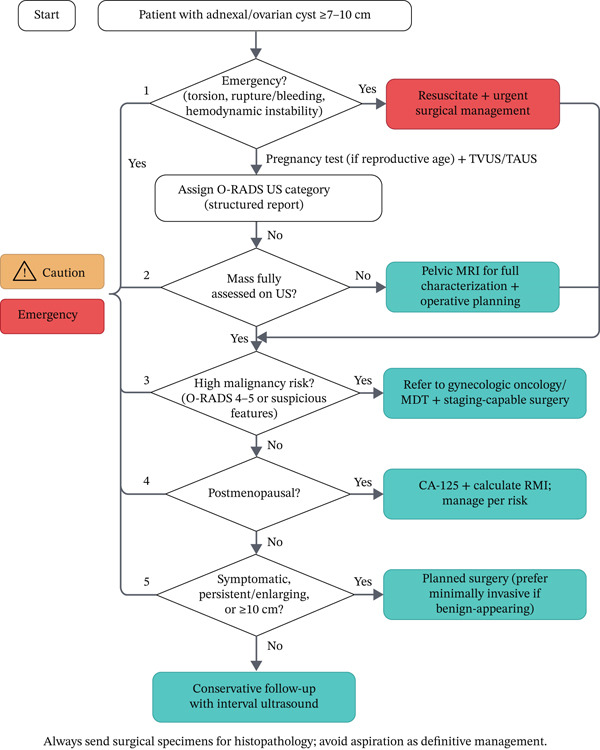
Management algorithm for large ovarian cysts (≥ 7–10 cm) using ultrasound risk stratification (O‐RADS) with escalation to MRI, surveillance, or surgery.

To support clinical decision‐making, a practical management algorithm for large ovarian cysts (≥ 7–10 cm) is provided (Figure 5), integrating structured ultrasound risk stratification and escalation to MRI, surveillance, or surgery based on symptoms, size, menopausal status, and malignancy‐risk features. The successful surgical management of this large ovarian cyst highlights the challenges involved in the treatment of such cases. The absence of intraoperative or postoperative complications validates this tailored surgical approach. This case reinforces that management of large ovarian cysts should not be algorithmic but must integrate patient preferences, reproductive goals, and detailed imaging findings to arrive at the safest and most effective surgical plan. This highlights the significance of precise imaging and surgical expertise in achieving a favorable result. The lack of fatal changes in this case further outlines the need for careful histopathological analysis in all examples of removal of ovarian cysts.

## 4. Conclusion

The case displays the successful, simple surgical management of a large benign ovarian cyst in a majority of women, which highlights the importance of comprehensive assessment and individualized management. Further research is needed to clarify optimal management strategies for large ovarian cysts, taking into account the patient′s factors, cyst characteristics, and potential long‐term results.

**Nomenclature**
BMI: body mass index
CS: C‐section
g/dL: grams per deciliter
Hb: hemoglobin
MRI: magnetic resonance imaging
O‐RADS: ovarian–adnexal reporting and data system
Plt: platelet
WBC: white blood cell

## Author Contributions

This is a collaborative work. All the authors significantly contributed to this study. In the preparation of this manuscript, the authors have made the following contributions: Dr. Ibrahim Talat Kasar provided surgical expertise, conceived the case report, and provided overall supervision and project administration. Dr. Elhadi Miskeen, Dr. Tagwa Idris, and Dr. Laila Yahya Alhubaishi offered critical oversight, validation of the clinical and radiological data, and thorough review and editing of the manuscript, with Dr. Laila Yahya Alhubaishi performing specific analysis of the MRI imaging.

## Funding

No funding was received for this manuscript.

## Disclosure

All authors have reviewed and provided final approval for the submitted version.

## Ethics Statement

Written informed consent was obtained from the patient for the publication of this case report, including the use of clinical data and imaging. All patient‐identifying information has been anonymized to protect confidentiality. The case report was prepared in accordance with the ethical standards of the institutional review board and the Helsinki Declaration.

## Conflicts of Interest

The authors declare no conflicts of interest.

## Data Availability

The data are not publicly available due to privacy or ethical restrictions.

## References

[1] Ackerman S. , Irshad A. , Lewis M. , and Anis M. , Ovarian Cystic Lesions: A Current Approach to Diagnosis and Management, Radiologic Clinics. (2013) 51, no. 6, 1067–1085, 10.1016/j.rcl.2013.07.010, 2-s2.0-84887180304, 24210445. 24210445

[2] Nweze S. O. , Abugu O. C. , Okolie E. I. , Ezenwaeze M. N. , Umekwe U. D. , Ortuanya K. E. , Awkadigwe F. I. , Enyinna P. K. , and Omeje C. U. , Normal Pregnancy Outcome After Emergency Ovarian Cystectomy for Ovarian Cyst Torsion in the First Half of Pregnancy: A Case Report, Journal of Surgical Research and Reviews. (2024) 1, no. 1, 10.5455/JSRR.20240730111452.

[3] Okugawa K. , Hirakawa T. , Fukushima K. , Kamura T. , Amada S. , and Nakano H. , Relationship Between Age, Histological Type, and Size of Ovarian Tumors, International Journal of Gynecology & Obstetrics. (2001) 74, no. 1, 45–50, 10.1016/S0020-7292(01)00406-4, 2-s2.0-0034955696. 11430940

[4] Melnyk M. , Starczewski A. , Nawrocka-Rutkowska J. , Gorzko A. , Melnyk B. , and Szydłowska I. , Giant Ovarian Tumors in Young Women: Diagnostic and Treatment Challenges—A Report of Two Cases and Narrative Review of the Recent Literature, Journal of Clinical Medicine. (2025) 14, no. 4, 10.3390/jcm14041236. PMC11856845 40004767

[5] Bašković M. , Habek D. , Zaninović L. , Milas I. , and Pogorelić Z. , The Evaluation, Diagnosis, and Management of Ovarian Cysts, Masses, and Their Complications in Fetuses, Infants, Children, and Adolescents, Healthcare. (2025) 13, no. 7, 10.3390/healthcare13070775, 40218072. PMC11988711 40218072

[6] Farghaly S. A. , Current Diagnosis and Management of Ovarian Cysts, Clinical and Experimental Obstetrics & Gynecology. (2014) 41, no. 6, 609–612, 10.12891/ceog20322014, 2-s2.0-84919772682, 25551948. 25551948

[7] Medeiros L. R. , Rosa D. D. , Bozzetti M. C. , Fachel J. M. , Furness S. , Garry R. , Rosa M. I. , Stein A. T. , and Cochrane Gynaecology and Fertility Group , Laparoscopy Versus Laparotomy for Benign Ovarian Tumour, Cochrane Database of Systematic Reviews. (2009) 2, 10.1002/14651858.CD004751.pub3, 2-s2.0-70349114709. PMC13590221 19370607

[8] D’Ambrosio V. , Brunelli R. , Musacchio L. , Del Negro V. , Vena F. , Boccuzzi G. , Boccherini C. , Di Donato V. , Piccioni M. G. , Benedetti Panici P. , and Giancotti A. , Adnexal Masses in Pregnancy: An Updated Review on Diagnosis and Treatment, Tumori Journal. (2021) 107, no. 1, 12–16, 10.1177/0300891620909144, 32180534. 32180534

[9] Farahani L. and Datta S. , Benign Ovarian Cysts, Obstetrics, Gynaecology & Reproductive Medicine. (2016) 26, no. 9, 271–275, 10.1016/j.ogrm.2016.06.003, 2-s2.0-84991074930.

[10] Mansour S. , Hamed S. , and Kamal R. , Spectrum of Ovarian Incidentalomas: Diagnosis and Management, The British Journal of Radiology. (2023) 96, no. 1142, 20211325, 10.1259/bjr.20211325. 35142537 PMC9975533

[11] Jarrell J. F. , The History of Gynecological Treatment of Women′s Pelvic Pain and the Recent Emergence of Pain Sensitization, 2024, Elsevier, 10.1016/B978-0-443-23994-6.00003-X.

[12] Oliveira I. J. , Pinto P. V. , and Bernardes J. , Noninvasive Diagnosis of Endometriosis in Adolescents and Young Female Adults: A Systematic Review, Journal of Pediatric and Adolescent Gynecology. (2025) 38, no. 2, 124–138, 10.1016/j.jpag.2024.07.005, 39098544. 39098544

[13] Lacy B. E. , Cangemi D. J. , Wise J. L. , and Crowell M. D. , Development and Validation of a Novel Scoring System for Bloating and Distension: The Mayo Bloating Questionnaire, Neurogastroenterology & Motility. (2022) 34, no. 8, e14330, 10.1111/nmo.14330, 35202489. 35202489

[14] Senarath S. , Ades A. , and Nanayakkara P. , Ovarian Cysts in Pregnancy: A Narrative Review, Journal of Obstetrics and Gynaecology. (2021) 41, no. 2, 169–175, 10.1080/01443615.2020.1734781. 32347749

[15] Jacobson J. A. , Middleton W. D. , Allison S. J. , Dahiya N. , Lee K. S. , Levine B. D. , Lucas D. R. , Murphey M. D. , Nazarian L. N. , Siegel G. W. , and Wagner J. M. , Ultrasonography of Superficial Soft-Tissue Masses: Society of Radiologists in Ultrasound Consensus Conference Statement, Radiology. (2022) 304, no. 1, 18–30, 10.1148/radiol.211101, 35412355. 35412355

[16] Garg V. and Gupta P. , Cystic Lymphangioma of the Gall Bladder: Common Disease at Uncommon Site, Egyptian Journal of Radiology and Nuclear Medicine. (2023) 54, no. 1, 10.1186/s43055-023-01103-z.

[17] Sinha A. and Ewies A. A. , Ovarian Mature Cystic Teratoma: Challenges of Surgical Management, Obstetrics and Gynecology International. (2016) 2016, no. 1, 2390178, 10.1155/2016/2390178, 2-s2.0-84984852056. 27110246 PMC4823513

[18] Murtani C. , Djohansjah A. O. , and Chandra S. , Giant Seromucinous Adenofibroma Ovarian Cyst in Nulliparous Woman: A Rare Case Report of Clinical Dilemma and Surgical Challenges, Journal of South Asian Federation of Obstetrics and Gynaecology. (2025) 17, no. 6, 860–864, 10.5005/jp-journals-10006-2794.

[19] Bogani G. , Borghi C. , Maggiore U. L. , Ditto A. , Signorelli M. , Martinelli F. , Chiappa V. , Lopez C. , Sabatucci I. , Scaffa C. , and Indini A. , Minimally Invasive Surgical Staging in Early-Stage Ovarian Carcinoma: A Systematic Review and Meta-Analysis, Journal of Minimally Invasive Gynecology. (2017) 24, no. 4, 552–562, 10.1016/j.jmig.2017.02.013, 2-s2.0-85015766607. 28223182

[20] Legrand C. , Keller L. , Collinet P. , Barbotin A. L. , Béhal H. , Rubod C. , and Decanter C. , Oocyte Accumulation for Fertility Preservation in Women With Benign Ovarian Tumours With a History of Previous Surgery, Multiple or Large Cysts, Reproductive Biomedicine Online. (2021) 43, no. 2, 205–214, 10.1016/j.rbmo.2021.04.020. 34247989

[21] Sadowski E. A. , Thomassin-Naggara I. , Rockall A. , Maturen K. E. , Forstner R. , Jha P. , Nougaret S. , Siegelman E. S. , and Reinhold C. , O-RADS MRI Risk Stratification System: Guide for Assessing Adnexal Lesions From the ACR O-RADS Committee, Radiology. (2022) 303, no. 1, 35–47, 10.1148/radiol.204371, 35040672. 35040672 PMC8962917

[22] American College of Obstetricians and Gynecologists , Practice Bulletin No. 174: Evaluation and Management of Adnexal Masses, Obstetrics and Gynecology. (2016) 128, no. 5, e210–e226, 10.1097/AOG.0000000000001768, 2-s2.0-85044957059, 27776072. 27776072

